# Evaluation of manual and automated approaches for segmentation and extraction of quantitative indices from [^18^F]FDG PET-CT images

**DOI:** 10.1088/2057-1976/ad160e

**Published:** 2024-01-05

**Authors:** Georgios Krokos, Tejas Kotwal, Afnan Malaih, Sally Barrington, Price Jackson, Rodney J Hicks, Paul K Marsden, Barbara Malene Fischer

**Affiliations:** 1 School of Biomedical Engineering and Imaging Sciences, King’s College London, London, United Kingdom; 2 Peter MacCallum Cancer Centre, Melbourne, Australia; 3 Department of Medicine, St Vincent’s Hospital Medical School, the University of Melbourne, Australia; 4 Dept. Clinical Physiology and Nuclear Medicine, Rigshospitalet, Copenhagen, Denmark; 5 Dept. of Clinical Medicine, University of Copenhagen, Denmark

**Keywords:** organ segmentation, PET, CT, atlas, deep learning

## Abstract

Utilisation of whole organ volumes to extract anatomical and functional information from computed tomography (CT) and positron emission tomography (PET) images may provide key information for the treatment and follow-up of cancer patients. However, manual organ segmentation, is laborious and time-consuming. In this study, a CT-based deep learning method and a multi-atlas method were evaluated for segmenting the liver and spleen on CT images to extract quantitative tracer information from Fluorine-18 fluorodeoxyglucose ([^18^F]FDG) PET images of 50 patients with advanced Hodgkin lymphoma (HL). Manual segmentation was used as the reference method. The two automatic methods were also compared with a manually defined volume of interest (VOI) within the organ, a technique commonly performed in clinical settings. Both automatic methods provided accurate CT segmentations, with the deep learning method outperforming the multi-atlas with a DICE coefficient of 0.93 ± 0.03 (mean ± standard deviation) in liver and 0.87 ± 0.17 in spleen compared to 0.87 ± 0.05 (liver) and 0.78 ± 0.11 (spleen) for the multi-atlas. Similarly, a mean relative error of −3.2% for the liver and −3.4% for the spleen across patients was found for the mean standardized uptake value (SUV_mean_) using the deep learning regions while the corresponding errors for the multi-atlas method were −4.7% and −9.2%, respectively. For the maximum SUV (SUV_max_), both methods resulted in higher than 20% overestimation due to the extension of organ boundaries to include neighbouring, high-uptake regions. The conservative VOI method which did not extend into neighbouring tissues, provided a more accurate SUV_max_ estimate. In conclusion, the automatic, and particularly the deep learning method could be used to rapidly extract information of the SUV_mean_ within the liver and spleen. However, activity from neighbouring organs and lesions can lead to high biases in SUV_max_ and current practices of manually defining a volume of interest in the organ should be considered instead.

## Introduction

Positron Emission Tomography (PET) imaging with fluorine-18 fluorodeoxyglucose ([^18^F]FDG) is widely used in the management of cancer patients [1]. In addition to evaluating tumour, the metabolic activity of uninvolved organs in the body may provide information regarding patient response, side-effects, and survival during anti-cancer treatment [2–4]. Moreover, established techniques such as the Deauville 5-point scale, directly utilise the [^18^F]FDG uptake in normal liver and mediastinum as reference regions, to evaluate treatment response in lymphoma [1]. Even for non-[^18^F]FDG studies, the assessment of tracer uptake in organs may provide valuable information regarding personalised treatment response and toxicity, such as in the emerging field of theranostics [5].

All of the examples mentioned above, require the delineation of multiple organs, or sections of the organs, on PET-CT images in order to measure the corresponding tracer uptake. The main barrier to achieve this, is the laborious and time-consuming process of manual segmentation and the lack of consensus around an optimal automatic method in clinic. Limitations in manual techniques and variability between observers and/or image quality led to the proposal of a framework for PET Response Criteria in Solid Tumours (PERCIST) using a spherical volume of interest (VOI) with 3-cm diameter, to measure quantitative or semi-quantitative uptake in liver [1, 6]. Even though this method can be quite robust in large organs with uniform uptake such as the liver and is less laborious compared to whole organ segmentation, it still requires some manual input especially in smaller organs in which a simpler 2D region of interest (ROI) on the appropriate transaxial slice is a more accurate approach [7]. Moreover, for applications where the whole volume of the region is of interest such as in dosimetry and theranostics, this method is inadequate.

Multi-atlas segmentation is widely applied for automatic organ segmentation in biomedical imaging. The main principle is to exploit prior knowledge from a database consisting of ‘labelled’ images to segment a new (target) image [8]. Most studies employing multi-atlas techniques focus on the brain which is less sensitive to registration errors between the atlases and the target image. However, a few studies have successfully applied multi-atlas techniques to thoracic and abdominal organs in CT, reporting a high degree of overlap with manual segmentation of the same images and the ability to adapt to large morphological differences between organs [8–12]. Moreover, many multi-atlas approaches are easily accessible in clinical settings as they are available through medical imaging software used for clinical reporting [13].

More recently, semantic segmentation using artificial intelligence approaches and specifically deep learning (DL), is gaining momentum. Contrary to the multi-atlas approach, minimal human intervention is required with a set of algorithms known as neural networks employed to extract underlying features from the labelled images. These provide information to enable segmentation of the investigated organ and the trained model can be rapidly applied to a new image [14]. Since no registration is required between the training dataset and the new image, this method has been broadly applied in CT segmentation of abdominal organs with studies reporting high similarity with manual segmentations [12, 15–22].

Even though most multi-atlas and deep learning studies have managed to generate highly accurate organ regions on CT images, the potential impact of transferring CT segmented regions to PET images for the evaluation of glucose metabolism has not been thoroughly investigated [23]. Moreover, in PET-CT imaging, it has been demonstrated that high accuracy in terms of Hounsfield Units from CT segmented organs does not always translate to accuracy in assessment of tracer uptake within organs [24]. The aims of this study were i) to compare the accuracy of a commercially available CT-based multi-atlas and a pre-trained deep learning method for segmenting the liver and spleen on CT images with manually drawn reference regions and ii) to assess the impact of manual and automated CT-based segmentation on estimation of [^18^F]FDG uptake in the two organs.

## Materials and methods

### Dataset

Baseline and interim [^18^F]FDG PET-CT scans from 50 UK patients who participated in the ‘Response-adapted therapy in patients with advanced Hodgkin’s lymphoma (RATHL)’ multi-centre trial were used for the analysis [1]. Only patients without major artefacts on the CT or PET images were included in the study. All scans used time-of-flight PET with ordered subset expectation maximisation reconstructions with low-dose CT acquired at 120 kVp or 140 kVp with current up to 160 mA for attenuation correction and anatomical localisation. All PET-CT scans used standardised procedures and were accredited by the UK PET Core lab [25]. All analyses were performed using MIM Maestro version 6.9.7, (MIM Software Inc., USA).

### Manual segmentations

Manual segmentations of the liver and spleen were performed separately on CT (CT-man) and PET (PET-man) images by a medical student and reviewed by a nuclear medicine physician with 15 years’ experience. In cases of disagreement, the two specialists would jointly assess the images before agreeing to the most appropriate regions. For the PET segmentation, the rigidly coregistered CT images were used for anatomical guidance and any metabolic activity from neighbouring organs that might affect standardised uptake values (SUVs) was excluded.

### Deep learning segmentation

A pre-trained 3D-UNET algorithm was applied to the CT images (CT-DL). Details regarding the network’s architecture and training can be found in [26]. In brief, the algorithm had been previously trained on 89 CT datasets from patients with manually segmented kidneys [26] and then retrained to include the liver and the spleen. The algorithm was trained in Keras (v2.08) using Python with Tensorflow backend (v1.3) in which slabs of 128 × 128 × 64 were provided for training and the dice-coefficient was used as the loss function. A new whole-body CT image undergoes rigid registration to a template CT image using the bony anatomy before it is cropped to a volume analogous to the organ of interest with approximate dimensions 334 × mm × 334 × mm × 320 mm which is approximately half of the of the size of the input images. The corresponding trained weights were applied to segment the organ.

Another deep learning model for segmenting CT images based on the nnU-NET algorithm called TotalSegmentator (CT-DL(TS)) which is publicly available and has demonstrated high accuracy as measured by the DICE coefficient [22], was also applied on the dataset.

### Multi-atlas-based segmentation

The multi-atlas library was generated by selecting a ‘template’ patient and registering the CT images of the template and the subjects comprising the multi-atlas list [13, 27]. The template patients for the liver and spleen were chosen to be representative of an ‘average-shaped’ patient. The CT images along with their respective manually segmented contours from 48 of the patients were brought to the template space using rigid registration. Visual inspection and fine-tuning was performed during the registration process if needed. The similarity indices between CT-man of the template and the rest of the patients were calculated and stored. To apply the multi-atlas on a target patient, the approximate area of the organ of interest was selected by the user from the whole-body CT image and the corresponding multi-atlas library applied. The similarity index was then calculated between template and target image and the patients with similar indices were identified. Finally, deformable registration between the library and target patient was performed to generate the new contour (CT-Atlas).

The libraries were created using the ‘leave-one-out’ cross-validation method [28]. Since the same template patient was used for all libraries for consistency, the atlases were only applied on the remaining patients.

### VOI definition

Spherical VOIs with 3-cm and 6-cm diameters (PET-VOI3 and PET-VOI6) were manually placed within the right lobe of the liver on the PET images avoiding the liver edge. PET-VOI3 was based on PERCIST criteria [6] whilst the larger PET-VOI6 (encompassed within the liver border) was selected as an approach potentially less sensitive to noise. For the spleen, only PET-VOI3 was used.

### Data analysis

Agreement between the manually and automatically segmented CT regions was evaluated using the DICE coefficient. All PET images were registered to the CT images using rigid registration and the CT-generated regions were transferred to the PET space using the inverse transformation matrix. The mean relative error (MRE) of the mean and maximum SUV (SUV_mean_, SUV_max_) in liver and spleen for: (1) CT-man, (2) CT-DL, (3) CT-Atlas, (4) PET-VOI3 and (5) PET-VOI6 (only for liver) was estimated using PET-man as reference.

The normality of the SUV differences between methods was assessed using the Kolmogorov-Smirnov test. The paired t-test and the non-parametric Wilcoxon-signed rank test were used to compare differences between PET-man and the various segmentation methods while Bland-Altman plots were used to assess agreement with confidence intervals set to 95%.

## Results

### Comparison of automated with manual CT segmentations

In ten patients, focal uptake was present in the spleen suggestive of lymphomatous involvement. These cases were excluded from subsequent analyses due to high ambiguity during annotation of the region.

**Figure 1.**
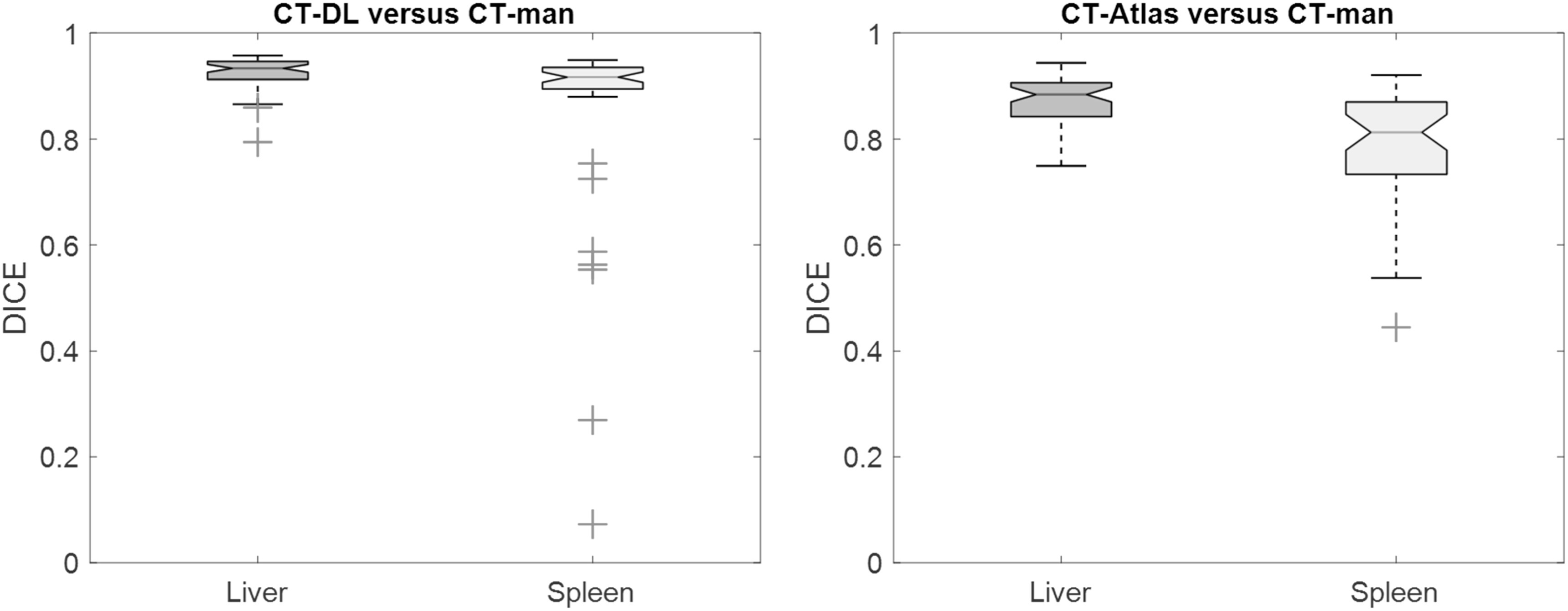
Box-plots with DICE scores of regions generated with CT-DL (left) and CT-Atlas (right) compared to CT-man for the liver and spleen. Outliers are indicated by red crosses.

**Figure 2.**
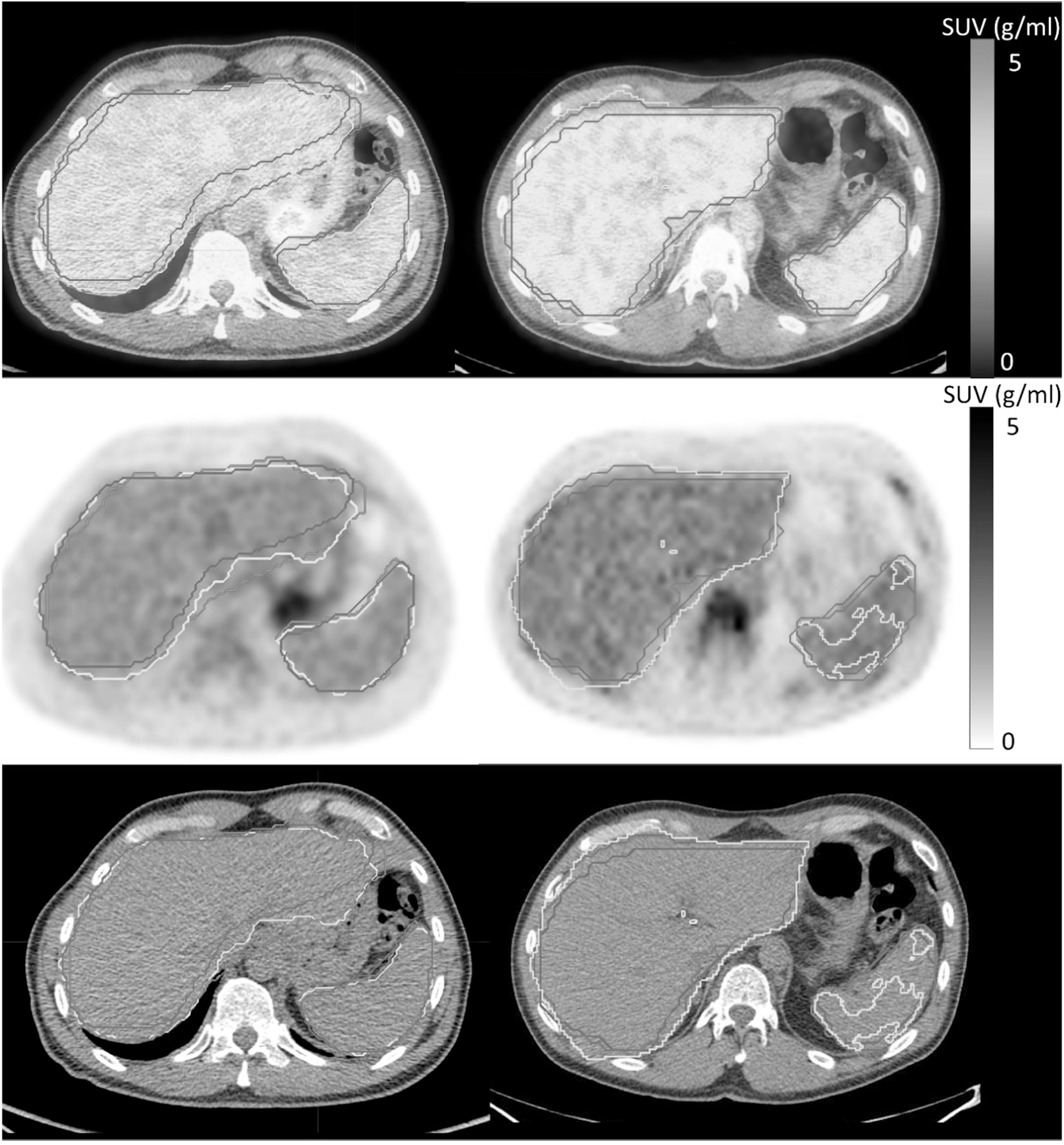
PET-CT fused images (first row) along with corresponding PET only (second row) and CT only images (third row) from two patients. The automated methods provided similar contours for the liver and spleen in patient 1 (left column) but failed to accurately segment organs in patient 2 (right column). The brown contour represents PET-man, the yellow CT-DL and the magenta the CT-Atlas.

A higher DICE coefficient of 0.93 ± 0.03 (mean ± std) was obtained for the CT-DL in liver compared to the DICE coefficient of 0.87 ± 0.05 for CT-Atlas. Similarly, the DICE coefficient for the spleen was 0.85 ± 0.19 for the CT-DL compared to 0.78 ± 0.11 for CT-Atlas. However, there were fewer outliers using the CT-Atlas than the CT-DL method (figure 1) with only a single outlier using the CT-atlas method, where part of the left lobe of the liver was misclassified as spleen. There were two outliers for the CT-DL regions for the liver where sections of enlarged livers were omitted and/or where regions extended to include parts of the lung, despite having relatively high DICE coefficients of 0.79 and 0.87 respectively. Most outliers using the CT-DL method for the spleen had large volumes of >300 ml where the method failed to accurately identify organ boundaries (figure 2).

CT-DL(TS) performed similarly in the liver as CT-DL, providing a DICE coefficient of 0.93 ± 0.03 but managed to better segment the enlarged spleens leading to a DICE coefficient of 0.92 ± 0.02 as shown in Suppl. figure 1.

### Comparison of manual CT with manual PET segmentations

**Figure 3.**
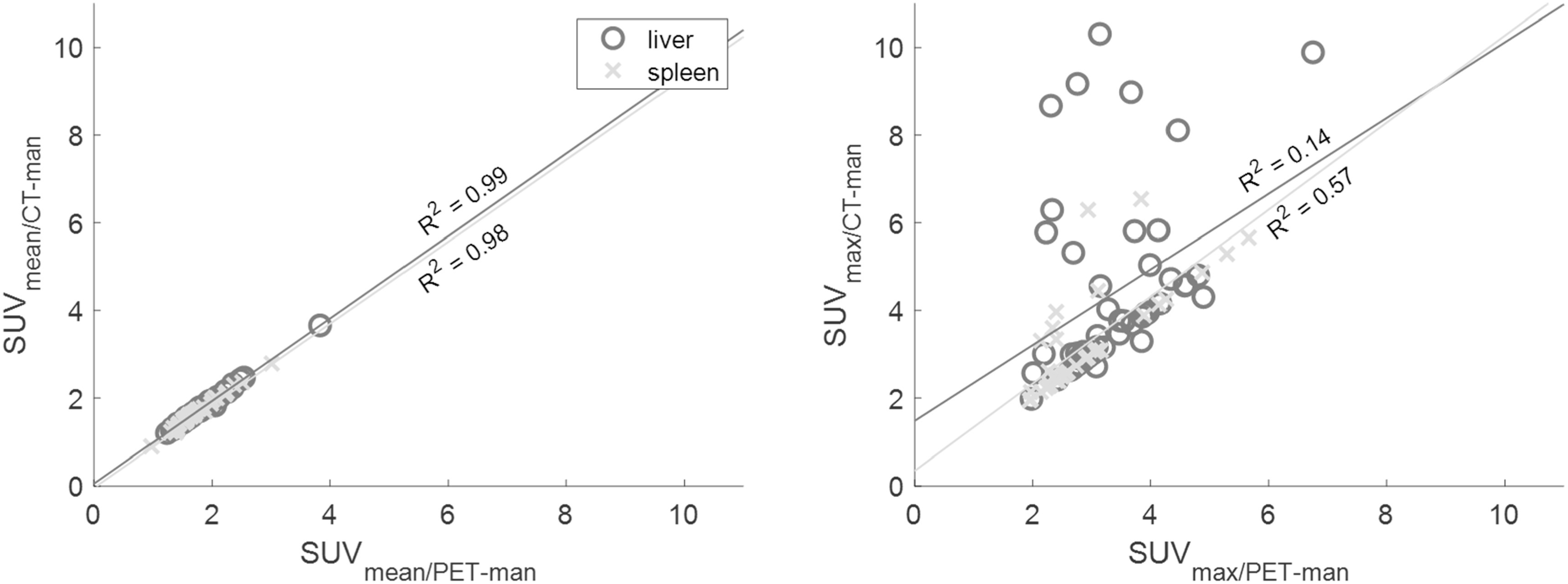
SUV_mean_ (left) and SUV_max_ (right) for manually segmented CT and PET regions in liver and spleen. The lines represent the linear fit for each dataset with the corresponding R^2^ value.

**Figure 4.**
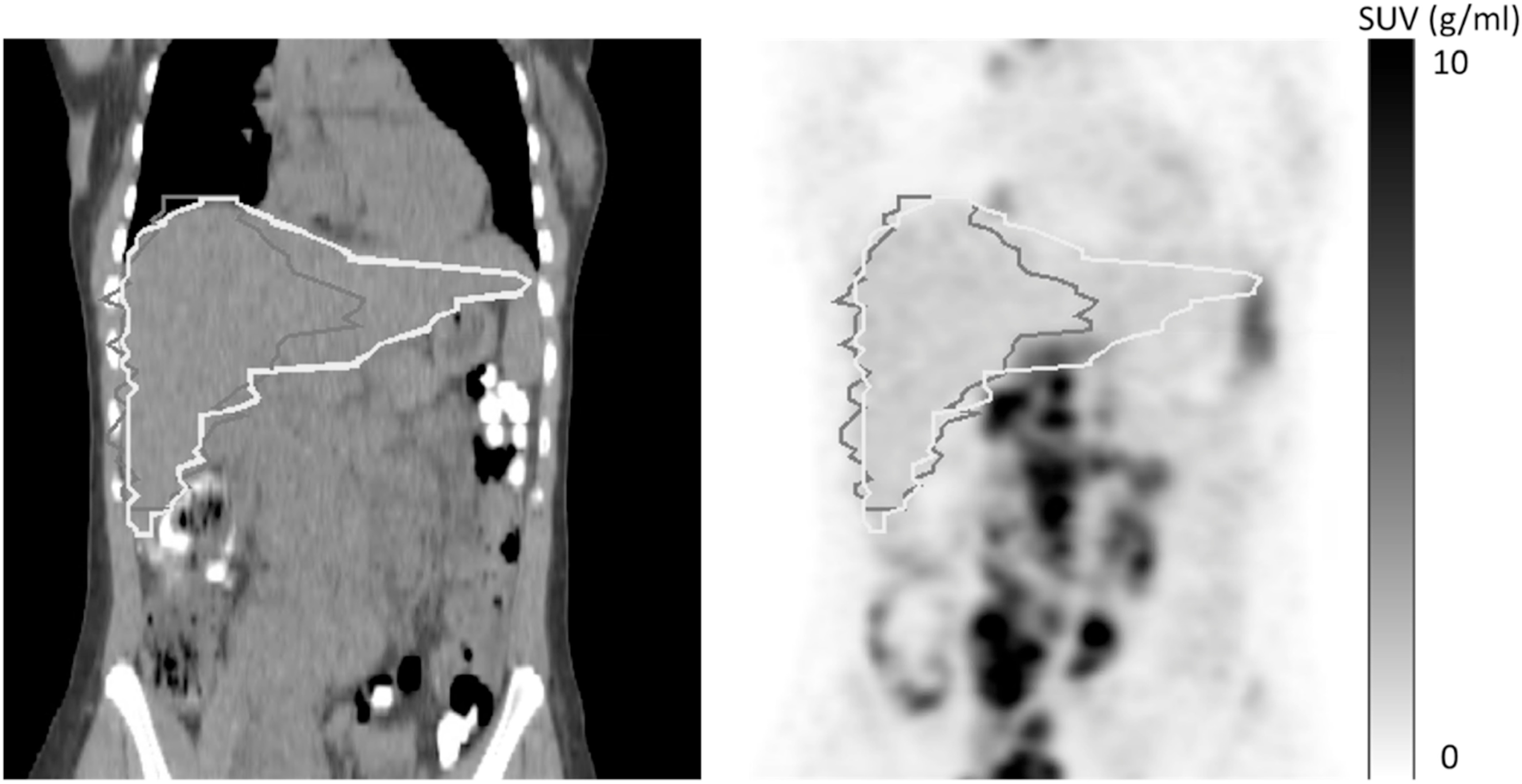
PET-man (brown) and CT-man (green) for the liver on a patient with poor agreement between the two techniques.

The CT-man and PET-man regions were similar with a DICE score of 0.87 ± 0.02 for the liver and 0.81 ± 0.05 for the spleen. The PET-man regions were however systematically smaller than CT-man with a difference in volume of 16 ± 9% (mean ± standard deviation) for the liver and 24 ± 21% for the spleen. Strong correlations were observed for SUV_mean_ in both organs (figure 3) and SUV_max_ in the spleen but not the liver, for which CT-man resulted in up to fourfold overestimation of SUV_max_. The CT-man region tended to overlap with the boundaries of neighbouring organs that had similar Hounsfield units such as the upper small and large intestine, abdominal nodes (figure 4) etc. All differences in SUV between PET-man and CT-man were statistically significant. Since CT-man resulted in bias when performing SUV_max_ analysis, only PET-man was used as reference to perform the comparisons in SUVs in the following sections.

### Comparison of automated CT segmentations and VOI methods with manual PET segmentation

**Figure 5.**
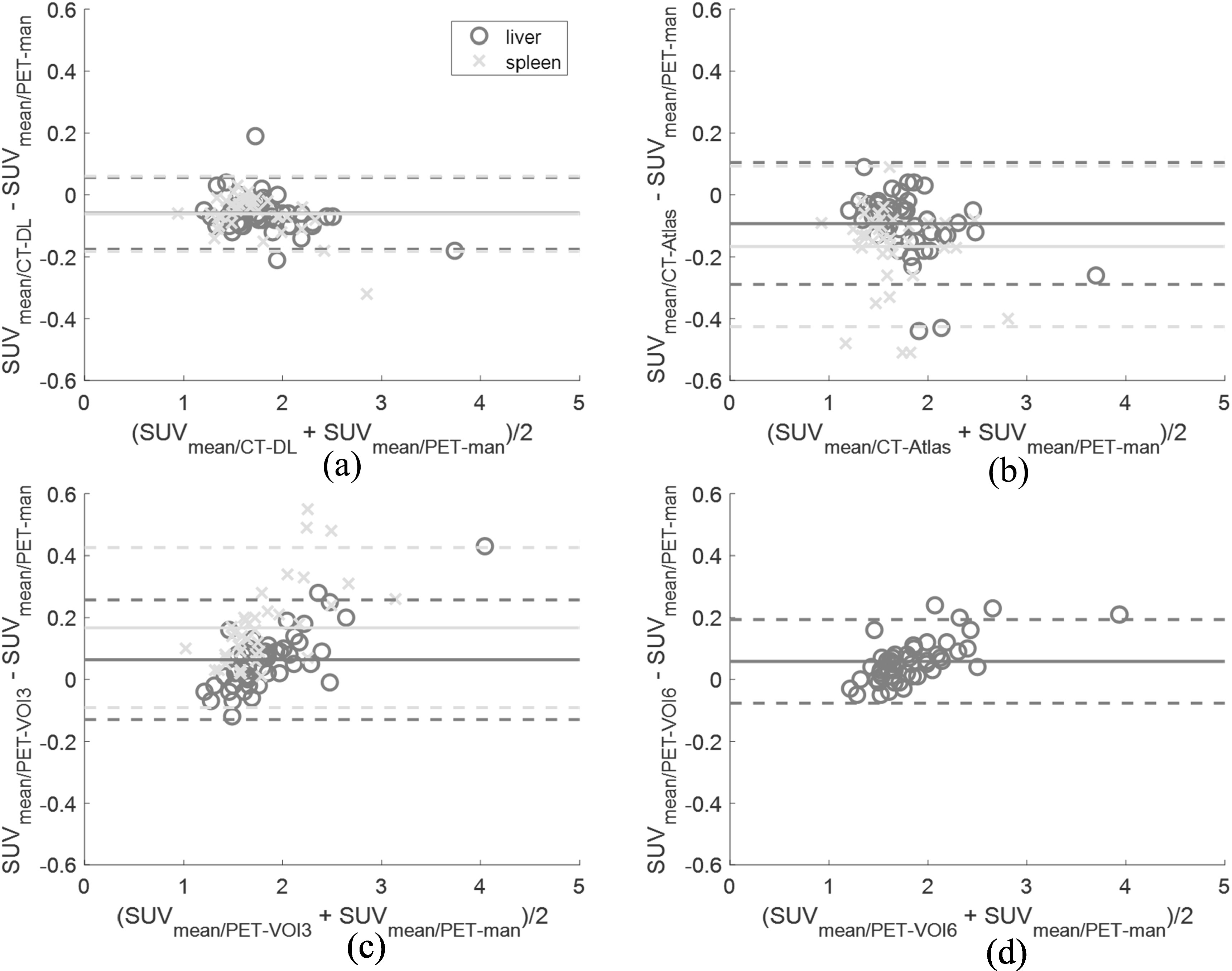
Bland-Altman plots for SUV_mean_ using (A) deep learning (top left), (B) multi-atlas method (top right). (C) 3-cm VOI (bottom left) and (D) 6-cm VOI (bottom right) compared to manually segmented PET regions in liver and spleen. Solid lines indicate the biases and dashed lines the 95% confidence intervals.

There was a small negative bias in SUV_mean_ for both organs using the automatic methods and a small positive bias using the VOI approaches compared to PET-man (figure 5). CT-DL was the most consistent across both organs with an absolute bias of 0.06 ± 0.06 g ml^−1^ which was comparable to the VOI methods (0.06 ± 0.10 g ml^−1^ and 0.06 ± 0.07 g ml^−1^ for PET-VOI3 and PET-VOI6, respectively) with 40% lower bias when compared to using CT-Atlas for liver segmentation. Furthermore, it outperformed the other methods for spleen segmentation with a > 2.5 times lower bias. CT-DL also provided the lowest standard deviation (figure 5 and table 1). Both VOI methods performed similarly with no statistically significant differences, although the larger 6-cm VOI addressed local inhomogeneity in the liver for one patient who appeared as an outlier using the 3-cm VOI for SUV_mean_. CT-DL(TS) provided similar results as CT-DL for SUV_mean_ with an absolute bias of 0.07 ± 0.04 g ml^−1^ in liver and 0.08 ± 0.09 g ml^−1^ in spleen (Suppl. figure 2).

**Table 1.** Average SUV_mean_ and SUV_max_ values for all methods and average mean relative errors compared to PET-man.

	SUV_mean_ (mean ± std)	MRE (%)	SUV_max_ (mean ± std)	MRE (%)	SUV_mean_ (mean ± std)	MRE (%)	SUV_max_ (mean ± std)	MRE (%)
	Liver (n = 50)				Spleen (n = 40)			
PET-man	1.82 ± 0.42		3.34 ± 0.90		1.71 ± 0.38		2.89 ± 0.89	
CT-man	1.77 ± 0.40	−2.8	4.37 ± 2.06	34.5	1.64 ± 0.36	−4.0	3.21 ± 1.17	11.7
CT-DL	1.76 ± 0.40	−3.2	6.89 ± 7.74	131.3	1.65 ± 0.35	−3.4	3.03 ± 0.89	6.9
CT-Atlas*	1.74 ± 0.39	−4.7	6.30 ±4.09	99.2	1.55 ± 0.36	−9.2	3.44 ± 1.67	23.2
PET-VOI3	1.89 ± 0.50	2.9	2.55 ± 0.67	−23.0	1.87 ± 0.46	9.3	2.58 ± 2.58	−10.6
PET-VOI6	1.88 ± 0.47	2.9	2.81 ± 0.68	−14.8				

* n = 49 for liver and n = 39 for spleen. The corresponding MREs are calculated for the same pair of patients.

**Figure 6.**
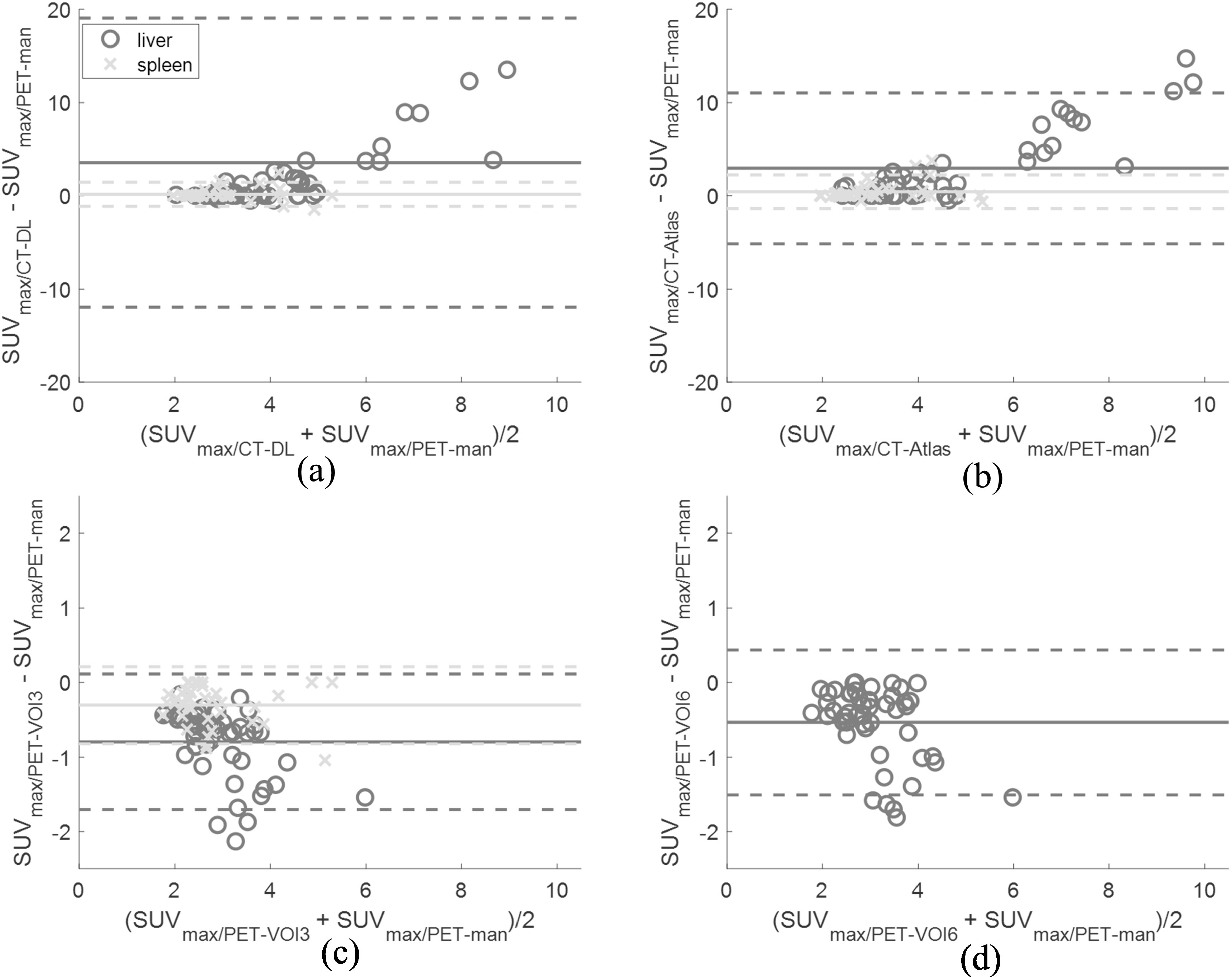
Bland-Altman plots for SUV_max_ using (A) deep learning (top left), (B) Multi-atlas method (top right) and (C) 3-cm VOI (bottom left) and (D) 6-cm VOI (bottom right) compared to manually segmented PET regions in liver and spleen. Solid lines indicate the biases and dashed lines the 95% confidence intervals. Note the difference in the axes limits for the automatic methods (top row). Two patients with a difference of over 29 g ml^−1^ in SUV_max_ are not displayed on the top left figure for visual clarity.

There were greater biases for measurement of SUV_max_ than SUV_mean_ with 5–23 fold higher average MREs in the liver for SUV_max_ (figure 6 and table 1). Larger biases, up to 3-fold higher were present in spleen.

**Figure 7.**
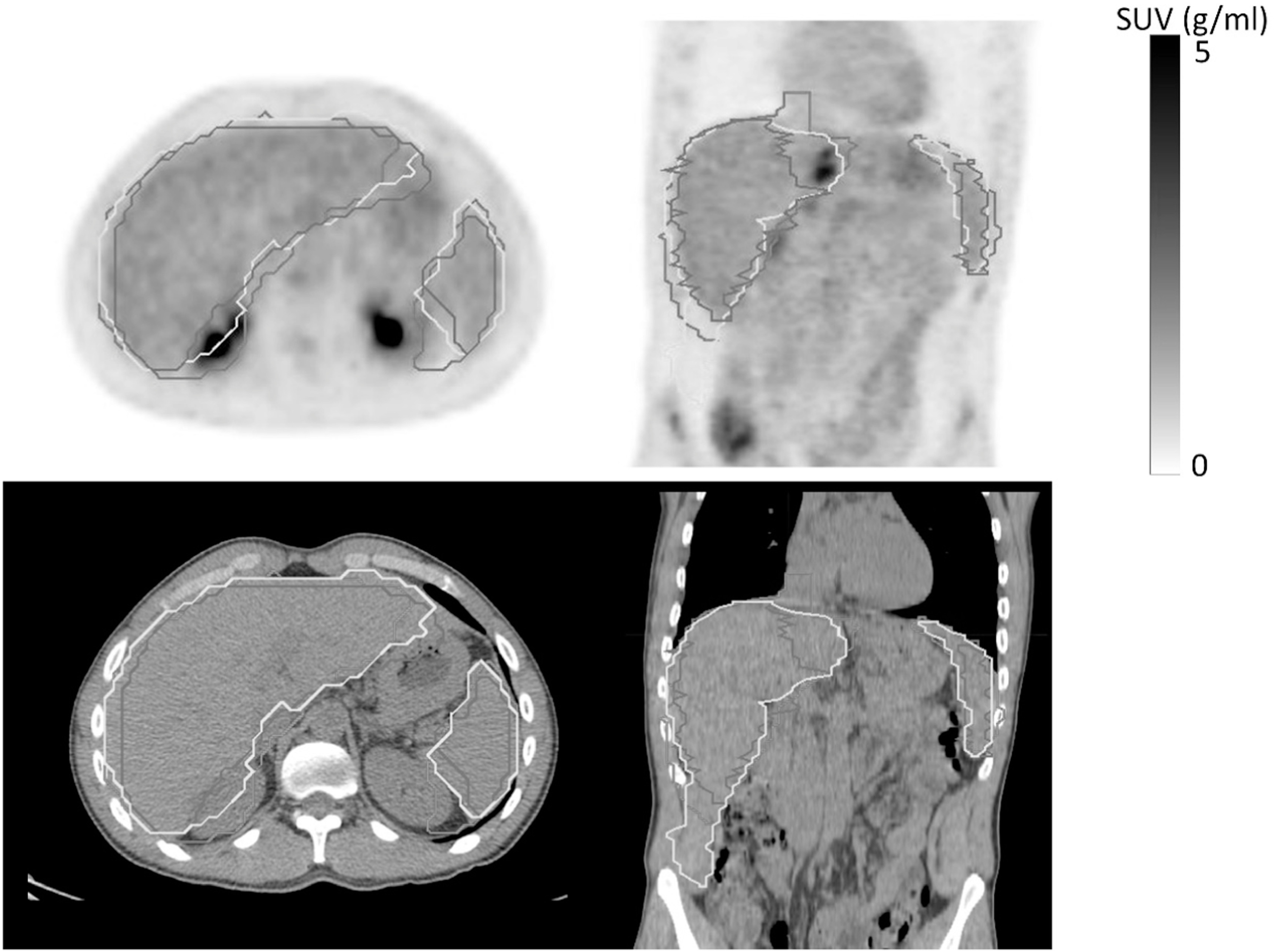
Axial (first column) and coronal (second column) view of PET (first row) and CT (second row) images a patient for which CT-DL and CT-Atlas had high DICE coefficient with PET-man but the extension of the regions to include right kidney led to high biases in SUV_max_. The brown contour represents PET-man, the yellow CT-DL and the magenta the CT-Atlas.

Twenty one CT-DL and 24 CT-Atlas liver regions included neighbouring lymphoma lesions and, in a few cases, myocardial and/or renal uptake leading to large positive biases in SUV_max_ (figure 6) with an average value of approximately 3.07 g/ml for both methods (see example patient in figure 7). This was halved when the CT-DL(TS) method was applied as shown in Suppl. figure 2. The more conservative VOI methods provided more accurate results with a bias of only −0.79 and −0.54 g ml^−1^ for PET-VOI3 and PET-VOI6, respectively. All methods performed better for spleen than liver with biases of 0.15 g ml^−1^ (CT-DL), 0.42 g ml^−1^ (CT-Atlas), −0.31 g ml^−1^ (PET-VOI3) and 0.27 g ml^−1^ (CT-DL(TS)).

The differences in SUV_mean_ and SUV_max_ between all methods and PET-man were statistically significant using either the paired t-test and the Wilcoxon signed-rank test with the exception of SUV_max_ in spleen estimated with CT-DL.

## Discussion

In this study the use of automatic methods, namely a multi-atlas and deep learning approach, for segmenting the liver and spleen on CT images and transferring the regions to the associated PET images for estimation of [^18^F]FDG SUV was evaluated and compared with more commonly used VOI methods. The main findings were that: (1) all methods provided comparable results for SUV_mean_, with the deep learning method being the most accurate, (2) both automated methods and the CT manual segmentations resulted in high biases for SUV_max_, and (3) the accuracy of the automatic methods depended on the organ of interest, with liver segmentation more often erroneously including neighbouring areas with high uptake than the spleen, leading to overestimation of SUV_max_ in a substantial proportion of patients.

The DICE coefficients for the multi-atlas method compared closely with previous reports that ranged between 0.77–0.94 for the liver and 0.60–0.90 for the spleen [9–12, 16, 29, 30]. Differences are likely due to variation in numbers and patient characteristics between studies, the quality of the CT images [10] and differences in methodology. It is possible a more sophisticated technique could improve our results, however, the method provided by MIM Maestro compares equally with, or may be better than, most commercially available software that provide multi-atlas segmentation [29–31].

We believe that our findings for the deep learning method can be generalised for most deep learning approaches which are using CT images for organ segmentation. As it was demonstrated in the results, the same conclusions are drawn even if a more widely implemented model such as TotalSegmentator is applied. The majority of the proposed methods are also based on a U-NET architecture and have been trained on low-dose CT images for non-cerebral organs similarly to the model used in this study providing comparable DICE scores, i.e. 0.93–0.97 for the liver [12, 15–17, 21, 22] and 0.90–0.97 for the spleen [15, 18, 19, 21, 22].

The main reason why the deep learning method (CT-DL) severely underestimated splenic volume for several patients was due to the splenic size being much larger than the reported normal range of 237 ± 77 ml [32], which is encountered in patients with HL. Such discrepancies have important clinical implications for measurement of spleen volume, for example in the assessment of drug effectiveness, surgical planning and other settings [33]. In contrast to CT-DL, which was trained on participants with normal sized organs, the multi-atlas method was developed in the patients included in this study and, therefore, was better at segmenting organs in some patients who were outliers using the CT-DL method. Moreover, a deep learning algorithm with a much larger and diverse training dataset such as TotalSegmentator managed to better segment the spleen in patients with splenomegaly. This emphasises the importance of carefully evaluating a method before clinical application, including testing of generalisability using external datasets and/or a subset of the population under study. However, the aim of this study was to evaluate the performance of a deep learning model trained on CT images for organ segmentation, when applied on [18 F]FDG images for SUV analysis. Therefore we refrained from fine-tuning the CT-DL algorithm or comparing it against multiple other deep learning and atlas approaches to identify the most accurate in terms of DICE coefficient.

The low bias in SUV_mean_ for both the deep learning and the multi-atlas methods was expected as uptake in the liver is relatively homogenous, meaning any discrepancies compared with the manual method were insufficient to lead to noticeable differences in liver SUV_mean_. Similarly for the spleen, although the CT-DL method consistently underestimated boundaries in patients with splenomegaly, because uptake was relatively uniform in the spleen, results for SUV_mean_ were comparable to the reference method. For the multi-atlas method on the other hand, segmentation sometimes extended to include neighbouring organs and lymphoma lesions with high SUVs, leading to higher relative errors especially for SUV_max_. The error in SUV_mean_ measurement affected the spleen more than the liver, due to the greater impact of uptake of voxels outside the organ on the proportionally smaller splenic volume. The accuracy of the VOI regions depended on the size of the region relative to the size of the organ as previously observed [7]. The 3-cm and 6-cm VOI resulted in similar measurements, although the error in SUV_mean_ was more pronounced using the 3-cm VOI in one patient. In general though, all methods performed reasonably well for all patients.

The patient cohort in this study was challenging as nearly a third of patients had lymphoma lesions or areas with high physiological uptake located close to the liver and spleen, meaning errors in segmentation affected the SUV, especially SUV_max_. Nonetheless, both deep learning and the atlas methods performed better in the smaller spleen for SUV_max_, with the deep learning methods being more accurate. As expected, the more conservative VOIs seemed to be a safer approach in this situation with the larger region (CT-VOI6) having higher chances of including the voxel of the highest SUV whilst avoiding overlap with regions outside the liver or spleen. In general, even though the multi-atlas and especially the deep learning methods seem promising for automated organ segmentation, this study indicates that visual inspection of the CT and PET images is needed prior to extracting the required information, particularly for SUV_max_.

Despite the errors in SUV_mean_ for all organs and SUV_max_ in the spleen being relatively small, the differences were statistically significant compared to the manual segmentations. However, the distinction between ‘statistical significance’ and of ‘practical importance’ needs to be made [34] as the absolute differences are within the repeatability of SUV measurement and are unlikely to affect clinical reporting [35].

The performance of the automated methods for extracting SUV depends on the accuracy of the manual segmentation on the CT images and therefore, it is not surprising that the SUV_max_ is poorly defined in liver since the manual CT segmentation also included neighbouring tissues in some patients. This suggests that inclusion of the functional information from the PET images in the development of the automatic method, might significantly improve the results. A few studies have suggested this approach, although a rigorous SUV assessment in organs unaffected by disease has not been performed [23, 36]. In such an approach, attention should be given on the annotated images that would be fed to the network. Since the manually segmented CT images could occasionally include neighbouring organs but also provide valuable information regarding the organ boundaries while the PET images can help further refine boundaries and avoid neighbouring lesions by using the functional information provided, both modalities should be consulted when defining the annotated regions. Moreover, the current study was performed on low-dose CT images which add an extra challenge both for the trained algorithms and the manual segmentation process in separating neighbouring organs with similar Hounsfield units.

Another point of consideration which becomes apparent in this study, is that when comparing regions although the DICE coefficient is an invaluable approach for investigating the similarity between two regions and potentially for improving a method, the applicability of a region depends on the scientific question. For example, despite the relatively high DICE coefficient between the manually segmented PET and CT regions, the two can only be used interchangeably if the SUV_mean_ is of interest but not the SUV_max_. A more constricted region instead, might have provided better accuracy for SUV_max_ but lower DICE coeffiecient.

In terms of practicality, the deep learning method was faster, taking less than 30 s to generate single organ outlines compared to 240 s for the multi-atlas. Even though the latter is still relatively quick, especially compared to laborious manual segmentation, it might be impractical if performed routinely for multiple organs. Additionally, the multi-atlas method is more user-dependant with the potential for greater variability in segmentation between users and imperfect registration between template and target patient affecting results [37].

One of the limitations of this study could be considered the number of patients comprising the multi-atlas library, however, previous studies have reported that no significant gains would be expected with a larger library [27, 38]. Different patient cohorts were used for training the deep learning algorithm and building the multi-atlas library, however the deep learning algorithms which were not trained on patients with HL still performed slightly better than the multi-atlas method. In general, the fact that the algorithms were tested on a challenging patient cohort, with scans acquired from multiple scanners with slightly different acquisition parameters increases the credibility of our results. Moreover, the methods were developed in a manner suitable for application in a clinical setting, were users can employ their own or commercially available software to create local atlases and/or a pre-trained deep learning algorithm to CT images. Finally, involuntary motion of the abdominal organs might have hampered edge detection in PET images. However, all images used in the study were deemed of sufficient quality for clinical reporting and the agreement of the registration of the organ between PET and CT was also visually inspected. The latter might have a bigger effect on regions with more pronounced motion e.g. lung and small lesions in which minor mis-registrations could lead to high discrepancies.

## Conclusion

Automatic methods using a commercially available multi-atlas and a U-NET based deep learning algorithm for segmenting the liver and the spleen on CT were feasible to measure mean tracer uptake on [^18^F]FDG PET images, with the deep learning method providing better accuracy. However, activity from neighbouring organs and lymphoma lesions can lead to high biases for maximum tracer uptake and manual outlining or manual editing of automated segmentation should be considered instead. The incorporation of functional information from the PET data in the segmentation process, could potentially improve accuracy in automatic organ segmentation.

## Data Availability

The data cannot be made publicly available upon publication due to legal restrictions preventing unrestricted public distribution. The data that support the findings of this study are available upon reasonable request from the authors.

## Disclosure

Authors have no conflict of interest to declare.

## Ethical statement

This study performed retrospective analysis on patients who participated in the Response Adapted Therapy in Advanced Hodgkin Lymphoma (RATHL) study. The patients had given written informed consent in accordance with the Declaration of Helsinki. The study had Human Ethics Committee approval in all participating countries. Clinicaltrials.gov no: NCT00678327.
